# Extravascular Hydrophobic Surfaces, Fat Droplets, and the Connection With Decompression Illness: Spinal, Joint Pain, and Dysbaric Osteonecrosis

**DOI:** 10.3389/fphys.2018.00305

**Published:** 2018-03-27

**Authors:** Ran Arieli

**Affiliations:** ^1^Israel Naval Medical Institute, IDF Medical Corps, Haifa, Israel; ^2^Eliachar Research Laboratory, Western Galilee Medical Center, Nahariya, Israel

**Keywords:** phospholipds, nanobubbles, lamellar bodies, diving, decompression bubbles

Decompression illness (DCI) may occur during a prolonged deep dive, after exiting the water, or following any other reduction in ambient pressure, due to the formation of bubbles from supersaturated gas. The most common symptom of DCI is pain in the region of the joints, accounting for 68% of all cases (Pollock and Buteau, 2017). One of the most serious type of DCI is that affecting the spinal cord. Another form of injury is dysbaric osteonecrosis, typically found in caisson and tunnel construction workers, commercial divers, diving fishermen, deep sea divers, and personnel engaged in submarine escape procedures.

Decompression bubbles can expand and develop only from pre-existing gas micronuclei. It is known that nanobubbles form spontaneously when a smooth hydrophobic surface is submerged in water containing dissolved gas. We have shown that these nanobubbles are the gas micronuclei underlying decompression bubbles and DCI (Arieli, 2017). We placed large ovine blood vessels in a cooled high pressure chamber at 1,000 kPa for about 20 h. After decompression, bubbles evolved at definite hydrophobic spots in all the types of blood vessel, henceforth termed “active hydrophobic spots” (AHS). The lung surfactant dipalmitoylphosphatidylcholine (DPPC) leaks from the lung and settles on blood vessels to form the AHS. Nanobubbles are formed on the surface of these oligolamellar layers of phospholipids, and on decompression expand into venous and arterial bubbles. We suggested that after releasing most of their gas in the lung, the venous bubbles are transformed into gas-containing microparticles which cause many of the symptoms of DCI.

However, nanobubbles may evolve on any other hydrophobic surface having an interface with aqueous tissue in the body. The body contains lipid droplets, mainly within adipocytes but also in many other cells. There is usually a phospholipid surfactant on the surface of the droplet that separates the hydrophobic from the aquatic domain, with the result that there is no hydrophobic-aqueous interface. However, the intermediates of metabolic reactions, such as unesterified sterols, diacylglycerol, or fatty acids, may accumulate on the droplet surface and loosen the continuum of phospholipids (Thiam et al., 2013). The droplet surface may also temporarily store hydrophobic proteins, and there are mechanisms by which phospholipids are removed to make the triacylglycerols available for the lipase. A discrepancy may appear during lipid loading between surface expansion and phospholipid production. Phospholipid lamellar bodies are produced from lipid droplets in a variety of cells (Schmitz and Müller, 1991). If there is a similar process leading to the appearance of nanobubbles at the interface of a soft lipid droplet and the aqueous surround, then a fat droplet with a gap in the phospholipid envelope could serve as a source of gas micronuclei. Nanobubbles on these lipid droplets could expand on decompression by gaining supersaturated gas from the surrounding tissue. A high nitrogen load will be available due to the high solubility of nitrogen in fat. Most of our adipose tissues are located in soft tissue, and may expand without causing injury. However, if fat droplets and other hydrophobic surfaces are contained within a rigid, confined structure such as bone marrow, joints or the spinal cord, their expansion would be harmful.

## Spinal cord decompression illness

Hills (1993) demonstrated large numbers of perivascular, oligolamellar phospholipid bodies in the white matter of the spinal cord. These phospholipids and hydrophobic proteins had a surface tension similar to that of lung surfactants. It is possible that lung surfactants leaked into the blood, settled on blood vessels within the spinal cord, and after blocking perfusion were excluded as extravascular bodies. However, lamellar bodies are also produced in macrophages during myelin regeneration, and after several weeks can be detected in the extracellular matrix (Schmitz and Müller, 1991). It may also be that within the myelin sheath, where DPPC is an essential element in the myelination process, nanobubbles are formed at some location where a hydrophobic lamella interfaces with an aqueous solution. Fragments may arise from the lateral loop of the myelin (Benjamins and Morell, 1978), which could then serve as such a location. Surfactant phospholipids and proteolipids are released from the myelin as bubbles evolve after decompression, which could point to myelin as the possible origin of the bubbles (Hills, 1994). These two sources of gas micronuclei could be responsible for the autochthonous bubbles in the white matter of the spinal cord (Francis et al., 1990), and for the bubbles within the myelin sheath (Sykes and Yaffe, 1985).

## Dysbaric osteonecrosis

Bone marrow is rich in lipids, which increase with age to between 50 and 70% of bone marrow volume in the adult (Cawthorn and Scheller, 2017). Osteonecrosis occurs almost exclusively in areas of predominantly fatty marrow (Sweet and Madewell, 1995). Nanobubbles on the surface of fat droplets within the bone marrow may be the nuclei from which bubbles expand, blocking the circulation and causing dysbaric osteonecrosis.

## Joint pain

Synovial surfactant is produced in the synovial membrane (type B synoviocytes). It forms an oligolamellar lining of phosphatidylcholine on the articular surface, which serves as a very effective boundary lubricant (Hills, 1989; Schwarz and Hills, 1996). As with the AHS, nanobubbles will be present on the membrane of the synovial space and become gas nuclei on decompression. It is also possible that a number of lipid droplets, either free in the synovial fluid or within neutrophils which become abundant after injury (Wise et al., 1987), have nanobubbles on their surface. On decompression, the expansion of these bubbles will cause pain in the joint. Different types of phospholipid are adsorbed by the surface of normal cartilage to act as a boundary lubricant (Sarma et al., 2001). Thus, nanobubbles may also be present on the cartilage side of the joint.

## Author contributions

The author confirms being the sole contributor to this work and approved it for publication.

### Conflict of interest statement

The author declares that the research was conducted in the absence of any commercial or financial relationships that could be construed as a potential conflict of interest.
